# A novel gyrovirus in a common pheasant (*Phasianus colchicus*) with poult enteritis and mortality syndrome

**DOI:** 10.1007/s00705-022-05417-7

**Published:** 2022-03-20

**Authors:** Enikő Fehér, Krisztina Bali, Eszter Kaszab, Katalin Ihász, Szilvia Jakab, Borbála Nagy, Krisztina Ursu, Szilvia L. Farkas, Krisztián Bányai

**Affiliations:** 1 Veterinary Medical Research Institute, Hungária krt 21, Budapest, 1143 Hungary; 2 Veterinary Diagnostic Directorate, National Food Chain Safety Office, Tábornok utca 2, Budapest, 1143 Hungary; 3 University of Veterinary Medicine, István utca 2, Budapest, 1078 Hungary

## Abstract

A novel gyrovirus was detected in an intestinal specimen of a common pheasant that died due to poult enteritis and mortality syndrome. The genome of the pheasant-associated gyrovirus (PAGyV) is 2353 nucleotides (nt) long and contains putative genes for the VP1, VP2, and VP3 proteins in an arrangement that is typical for gyroviruses. Gyrovirus-specific motifs were identified in both the coding region and the intergenic region of the PAGyV genome. The VP1 of PAGyV shares up to 67.6% pairwise nt sequence identity with reference sequences and forms a distinct branch in the phylogenetic tree. Thus, according to the recently described species demarcation criteria, PAGyV belongs to a novel species in the genus *Gyrovirus*, family *Anelloviridae*, for which we propose the name "*Gyrovirus phaco 1*".

The genus *Gyrovirus* of the family *Anelloviridae* consists of viruses with a negative-sense, single-stranded, circular DNA genome, ~2.2–3.6 kilobases (kb) in length [1–12]. The genome of gyroviruses typically contains three main overlapping open reading frames (ORFs) that encodes structural (VP1) and non-structural regulatory proteins (VP2 and VP3) [1–12].

Gyroviruses are classified into 10 species, including *Chicken anemia virus* and nine additional species: *Gyrovirus fulgla 1*, *Gyrovirus galga 1* and *2*, *Gyrovirus homsa 1*, *2*, *3* and *4*, *Gyrovirus hydho1*, and *Gyrovirus myferr 1* [13]. Chicken anemia virus (CAV) is an immunosuppressive agent of chickens that can cause growth and feathering abnormalities, as well as anemia, and predisposes the host to secondary infections [14, 15]. Other gyroviruses have been identified in organ and fecal specimen of domestic and wild birds (e.g., chicken*, Gallus gallus*; northern fulmar, *Fulmarus glacialis*; crested screamer, *Chauna torquata*; ashy storm petrel, *Hydrobates homochroa*; ferruginous-backed antbird, *Myrmoderus ferrugineus*; white-plumed antbird, *Pithys albifrons*; grey teal, *Anas gracilis*; pigeon, *Columba livia*; Pekin duck, *Anas platyrhynchos*), mammals (human, *Homo sapiens*; domestic cat, *Felis catus*; ferret, *Mustela putorius furo*) and reptiles (king rat snake, *Elaphe carinata*) [1–13, 16–20]. Although there is no evidence that these viruses are pathogenic to their respective hosts, a recent study described gyrovirus 3 (GyV3, species *Gyrovirus homsa 1*) to be a multi-host pathogen, infecting mice and chickens [21].

In this study, a mixed organ sample (intestine, brain, heart, liver, and spleen) of a common pheasant (*Phasianus colchicus*) was subjected to metagenomic analysis. The bird succumbed to poult enteritis and mortality syndrome in 2017 on a pheasant farm in Hungary. Approximately 100 mg of specimen was homogenized in phosphate-buffered saline (PBS), using a TissueLyzer LT instrument (QIAGEN, Hilden, Germany). The homogenate was centrifuged (10,000 × *g* for 5 min) and filtered through a 0.45-µm PES filter. Nucleic acid was extracted using a NucleoSpin RNA Virus Kit (Macherey-Nagel, Düren, Germany). After amplification by random RT-PCR, a cDNA library was prepared for next-generation sequencing on an Illumina NextSeq™ 500 platform according to a previously described protocol [22]. The trimmed reads were submitted for taxonomic classification to the Kaiju web server [23]. Sequence reads were assembled *de novo* into contigs using Geneious Prime software v.2020.2.4 (Biomatters, Auckland, New Zealand) and were checked by BLAST analysis. The sequences were edited and aligned using AliView and Geneious Prime software [24]. After *de novo* assembly, a missing sequence in the non-translated region (NTR) of the genome was obtained by direct sequencing of two PCR products. Twenty μl of PCR reaction mixture, prepared for amplification of this region, contained 250 μM dNTPs, 250 nM primers (GyV3-F1 [5’-ACACGGAGAAATCCTGGTAAAC-3’] and GyV3-R1 [5’-ACTTAGTGTACACGTCTCGAGA-3’]; GyV3-F2 [5’-AGATAGACTCCATTTGGCAACTG-3’] and GyV3-R2 [5’-TGAGAATGACCACGCGTATAC-3’]), 1x DreamTaq Buffer, 0.625 U of DreamTaq DNA Polymerase (Thermo Fisher Scientific, Waltham, MA, USA) and 1 μL of the nucleic acid. The PCR thermal profile consisted of an initial denaturation step at 95 °C for 3 min, 45 cycles of amplification with steps of 95 °C for 30 s, 53 °C for 30 s, and 72 °C for 1 min, followed by a final elongation step at 72 °C for 10 min. The ORFs of the assembled viral genome sequence were predicted using the ORF Finder tool (https://www.ncbi.nlm.nih.gov/orffinder/). Phylogenetic analysis of the VP1 coding region was performed by the maximum-likelihood method (GTR+G model, 1000 bootstrap replicates), using MEGA6 software [25]. Pairwise identity values were calculated using SDTv1.2 and Geneious Prime software [26]. Recombination analysis was carried out using RDP4 software, utilizing representative complete genome sequences of gyroviruses [27]. Nuclear localization signals (NLS) were predicted using NLStradamus and cNLS Mapper, while nuclear export signals (NES) were identified using the online tool NetNES [28–30]. Amino acid motifs were identified using Motif Scan (https://myhits.sib.swiss/cgi-bin/motif_scan). Sequence repeats were found using Repeat Finder, implemented in the Geneious Prime software (Biomatters, Auckland, New Zealand).

Altogether, 17,507,704 reads were generated, and the Kaiju metagenomics pipeline revealed traces of gyrovirus sequences. A total of 3,653,320 sequence reads mapped to a 2353-nucleotide (nt)-long *de novo*-assembled viral genome sequence with a mean sequencing depth of 223,521X (range, 79X to 662,901X) (Fig. 1).

**Fig. 1:**
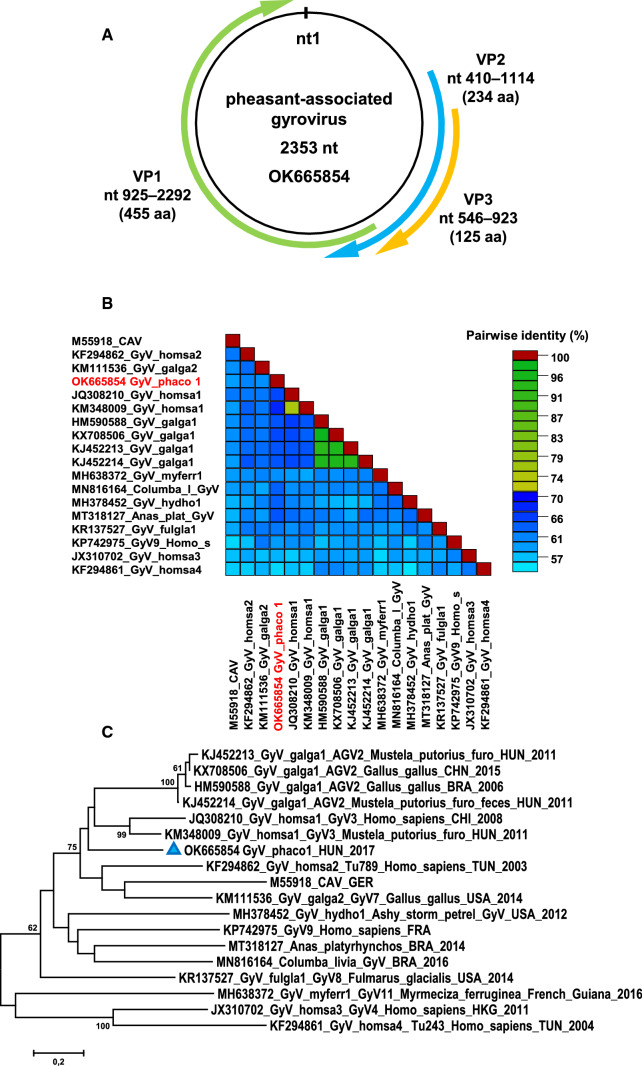
(A) Schematic representation of the pheasant-associated gyrovirus (PAGyV) genome. (B) VP1-nucleotide-sequence-based pairwise identity matrix of representative gyrovirus sequences, made using SDT v1.2 software. (C) Unrooted maximum-likelihood phylogenetic tree of representative gyrovirus VP1 nucleotide sequences. Support values less than 60 are hidden. PAGyV is indicated by a blue triangle.

The genome of the pheasant-associated gyrovirus (PAGyV) shows ≤67.1% genome-wide sequence identity with its closest relatives, GyV3 strains. Three major ORFs, encoding the VP1, VP2, and VP3 proteins, were identified in the PAGyV genome. The PAGyV VP1 shares up to 67.6% nt and 67.3% aa pairwise identity with the cognate genomic region of GyV3 and avian gyrovirus 2 (AGV2, species *Gyrovirus galga 1*) (Fig. 1). The common branch in the VP1-nt-based phylogenetic tree confirmed that GyV3 and AGV2 strains are the closest relatives of PAGyV (Fig. 1C). The sequences of PAGyV showed a maximum of 77.9% nt and 69.2% aa pairwise identity in VP2 and 69.2% nt and 59.4% aa identity in VP3, with GyV3 and AGV2 reference sequences. No recombination events were identified in the PAGyV sequence.

The identification of NLS and NES motifs in VP1 of gyroviruses implies that virion assembly occurs in the cell nucleus [11, 12, 31]. The N-terminal part of this protein in PAGyV is rich in arginine and may include an NLS between aa 6 and aa 52. Although leucine- and isoleucine- rich regions were detected in the VP1 of PAGyV, the signal finder tools did not identify any export signals, which may be a consequence of motif variations in the PAGyV sequence. Like other members of the family *Anelloviridae*, PAGyV likely produces nascent genomic DNA by rolling-circle replication, mediated by the putative rolling-circle replication motifs I, II, and III (^325^FATLSALG^332^, ^362^GRRWMTLVP^370^, and ^391^ATLFLAQG^398^, respectively) in VP1. Although the VP1 proteins of members of different species may be distantly related, conserved aa sites could be identified in addition to the above-mentioned motifs as parts of probable common functional motifs, e.g., ^58^PGXXXVRXXXP^68^, ^76^FXG^78^, ^130^GGP^132^, ^172^WWXW^175^, ^228^XXXXASLXXQXXY^240^, and ^348^XFNXHXXXGXXDP^359^ [1, 4–8, 11, 12].

The VP2 and VP3 proteins of gyroviruses are believed to be non-structural proteins that play a role in viral replication and pathogenesis [32, 33]. The VP2 of PAGyV contains a conserved WX_7_HX_3_CXCX_5_H protein tyrosine phosphatase motif (at aa position 102–122) that may be responsible for phosphorylation of a tyrosine residue in the C-terminal region of the VP3 protein [1, 4–8, 11, 32]. An NLS motif (^153^RAKRKLDYWKRKPKKPK^169^) was also predicted in the central part of VP2. The virus-encoded apoptin (the VP3 protein) induces apoptosis of erythroblastoid cells and thymocytes in CAV-infected chickens [32]. The VP3 aa sequence of PAGyV, like those of CAV and other gyroviruses, contains a potential NLS (^86^SPPRPRR^92^) as well as a putative leucine- and isoleucine-rich NES region (^42^ILIGIGSTTIELSL^55^) [11, 12, 32]. However, unlike other gyroviruses, the motif finder tools did not give strong support for a bipartite NLS, NES, or a tyrosine phosphorylation site in the C-terminal region of the VP3 of PAGyV.

The 470-nt-long NTR, located between the 3’ end of the VP1 gene and the 5’ end of the VP2 gene, contains a GC-rich region that may form loops, an AATAAA polyadenylation signal (at nt 80-85), and one copy of the 21-nt-long gyrovirus-specific promoter-enhancer sequence (TGTACAGGGGGGTACGTCA), which is preceded by three repeats of its complementary sequence (TGACGTACCCCCCTGTACA). The number and direction of these repeats seem to vary among gyroviruses [1, 4–8, 11, 12]. The NTR of the PAGyV genome shares the highest similarity with those of GyV3 isolates.

Gyrovirus taxonomy has been revised recently [13]. The calculated pairwise identity values (<69% for the VP1 coding gene), together with phylogenetic analysis, suggested that PAGyV is the first member of a putative new gyrovirus species. According to the suggested nomenclature for gyrovirus species, in which the initial letters of the scientific name of the host species are used, we propose the name "*Gyrovirus phaco 1*" for this tentative species. Given that gyroviruses may infect multiple hosts, the true host range of PAGyV and the potential role of this virus in poult enteritis and mortality syndrome need to be examined in future studies.

## Data Availability

The sequence data are available in the GenBank database with accession number OK665854.

## References

[1] Cibulski S, Weber MN, de Sales Lima FE, Lima DA, Fernandes Dos Santos H, Teixeira TF, Varela APM, Tochetto C, Mayer FQ, Roehe PM (2020). Viral metagenomics in Brazilian Pekin ducks identifies two gyrovirus, including a new species, and the potentially pathogenic duck circovirus. Virology.

[2] Fehér E, Pazár P, Kovács E, Farkas SL, Lengyel G, Jakab F, Martella V, Bányai K (2014). Molecular detection and characterization of human gyroviruses identified in the ferret fecal virome. Arch Virol.

[3] Fehér E, Pazár P, Lengyel G, Phan TG, Bányai K (2015). Sequence and phylogenetic analysis identifies a putative novel gyrovirus 3 genotype in ferret feces. Virus Genes.

[4] Li L, Pesavento PA, Gaynor AM, Duerr RS, Phan TG, Zhang W, Deng X, Delwart E (2015). A gyrovirus infecting a sea bird. Arch Virol.

[5] Loiko MR, Varela APM, Tochetto C, Lopes BC, Scheffer CM, Morel AP, Vidaletti MR, Lima DA, Cerva C, Mayer FQ, Roehe PM (2020). Novel Gyrovirus genomes recovered from free-living pigeons in Southern Brazil. Virology.

[6] Phan TG, Li L, O'Ryan MG, Cortes H, Mamani N, Bonkoungou IJO, Wang C, Leutenegger CM, Delwart E (2012). A third gyrovirus species in human faeces. J Gen Virol.

[7] Phan TG, Phung Vo N, Sdiri-Loulizi K, Aouni M, Pothier P, Ambert-Balay K, Deng X, Delwart E (2013). Divergent gyroviruses in the feces of Tunisian children. Virology.

[8] Phan TG, da Costa AC, Zhang W, Pothier P, Ambert-Balay K, Deng X, Delwart E (2015). A new gyrovirus in human feces. Virus Genes.

[9] Truchado DA, Diaz-Piqueras JM, Gomez-Lucia E, Doménech A, Milá B, Pérez-Tris J, Schmidt-Chanasit J, Cadar D, Benítez L (2019). A novel and divergent gyrovirus with unusual genomic features detected in wild passerine birds from a remote rainforest in French Guiana. Viruses.

[10] Waits K, Bradley RW, Warzybok P, Kraberger S, Fontenele RS, Varsani A (2018). Genome sequence of a gyrovirus associated with ashy storm-petrel. Microbiol Resour Announc.

[11] Rijsewijk FA, Dos Santos HF, Teixeira TF, Cibulski SP, Varela AP, Dezen D, Franco AC, Roehe PM (2011). Discovery of a genome of a distant relative of chicken anemia virus reveals a new member of the genus Gyrovirus. Arch Virol.

[12] Sauvage V, Cheval J, Foulongne V, Gouilh MA, Pariente K, Manuguerra JC, Richardson J, Dereure O, Lecuit M, Burguiere A, Caro V, Eloit M (2011). Identification of the first human gyrovirus, a virus related to chicken anemia virus. J Virol.

[13] Kraberger S, Opriessnig T, Celer V, Maggi F, Okamoto H, Blomström AL, Cadar D, Harrach B, Biagini P, Varsani A (2021). Taxonomic updates for the genus *Gyrovirus* (family *Anelloviridae*): recognition of several new members and establishment of species demarcation criteria. Arch Virol.

[14] Fatoba AJ, Adeleke MA (2019). Chicken anemia virus: a deadly pathogen of poultry. Acta Virol.

[15] Yuasa N, Taniguchi T, Yoshida IN, Yuasa T, Taniguchi IY (1979). Isolation and some characteristics of an agent inducing anemia in chicks. Avian Dis.

[16] Cibulski S, Alves de Lima D, Fernandes Dos Santos H, Teixeira TF, Tochetto C, Mayer FQ, Roehe PM (2021). A plate of viruses: Viral metagenomics of supermarket chicken, pork and beef from Brazil. Virology.

[17] Goldberg TL, Clyde VL, Gendron-Fitzpatrick A, Sibley SD, Wallace R (2018). Severe neurologic disease and chick mortality in crested screamers (*Chauna torquata*) infected with a novel Gyrovirus. Virology.

[18] Niu JT, Yi SS, Dong GY, Guo YB, Zhao YL, Huang HL, Wang K, Hu GX, Dong H (2019). Genomic characterization of diverse gyroviruses identified in the feces of domestic cats. Sci Rep.

[19] Vibin J, Chamings A, Klaassen M, Alexandersen S (2020). Metagenomic characterisation of additional and novel avian viruses from Australian wild ducks. Sci Rep.

[20] Wu Q, Xu X, Chen Q, Ji J, Kan Y, Yao L, Xie Q (2019). Genetic Analysis of avian gyrovirus 2 variant-related gyrovirus detected in farmed king ratsnake (*Elaphe carinata*): the first report from China. Pathogens.

[21] Yuan S, Yan T, Huang L, Hao X, Zhao M, Zhang S, Zhou D, Cheng Z (2021). Cross-species pathogenicity of gyrovirus 3 in experimentally infected chickens and mice. Vet Microbiol.

[22] Bali K, Bálint Á, Farsang A, Marton S, Nagy B, Kaszab E, Belák S, Palya V, Bányai K (2021). Recombination events shape the genomic evolution of infectious bronchitis virus in Europe. Viruses.

[23] Menzel P, Ng KL, Krogh A (2016). Fast and sensitive taxonomic classification for metagenomics with Kaiju. Nat Commun.

[24] Larsson A (2014). AliView: a fast and lightweight alignment viewer and editor for large datasets. Bioinformatics.

[25] Tamura K, Stecher G, Peterson D, Filipski A, Kumar S (2013). MEGA6: molecular evolutionary genetics analysis version 6.0. Mol Biol Evol.

[26] Muhire BM, Varsani A, Martin DP (2014). SDT: A virus classification tool based on pairwise sequence alignment and identity calculation. PLoS ONE.

[27] Martin DP, Murrell B, Golden M, Khoosal A, Muhire B (2015). RDP4: Detection and analysis of recombination patterns in virus genomes. Virus Evol.

[28] Kosugi S, Hasebe M, Tomita M, Yanagawa H (2009). Systematic identification of yeast cell cycle-dependent nucleocytoplasmic shuttling proteins by prediction of composite motifs. Proc Natl Acad Sci USA.

[29] Nguyen Ba AN, Pogoutse A, Provart N, Moses AM (2009). NLStradamus: a simple Hidden Markov Model for nuclear localization signal prediction. BMC Bioinformatics.

[30] la Cour T, Kiemer L, Mølgaard A, Gupta R, Skriver K, Brunak S (2004). Analysis and prediction of leucine-rich nuclear export signals. Protein Eng Des Sel.

[31] Cheng JH, Lai GH, Lien YY, Sun FC, Hsu SL, Chuang PC, Lee MS (2019). Identification of nuclear localization signal and nuclear export signal of VP1 from the chicken anemia virus and effects on VP2 shuttling in cells. Virol J.

[32] Feng C, Liang Y, Teodoro JG (2020). The role of apoptin in chicken anemia virus replication. Pathogens.

[33] Peters MA, Jackson DC, Crabb BS, Browning GF (2002). Chicken anemia virus VP2 is a novel dual specificity protein phosphatase. J Biol Chem.

